# Supplementary material for: Association Between Early Point-of-Care Ultrasound and Emergency Department Outcomes in Admitted Patients with Non-Traumatic Abdominal Pain: A Propensity Score-Weighted Cohort Analysis
Source: Diagnostics (Basel). 2025 Dec 12;15(24):3182. doi: 10.3390/diagnostics15243182 (PMC12732229; doi:10.3390/diagnostics15243182)
Supplement: Supplementary file 1 [file diagnostics-15-03182-s001.zip › diagnostics-3997226-supplementary.pdf]

Supplementary Table S1. Baseline characteristics and covariate balance before and after IPTW for the sensitivity analysis comparing the excluded 'PoCUS > 1 hour' cohort (N=864) and the 'No PoCUS' cohort (N=5,324)

	Data before IPTW			Data after IPTW		
	No-PoCUS N=5324 (86.04)	PoCUS > 1 hr N=864 (13.96)	p	No-PoCUS	PoCUS > 1 hr	SMD
Age	58.00 (42.00 to 71.00)	56.00 (40.00 to 70.00)	0.008	58.00 (42.00 to 71.00)	58.00 (42.00 to 71.00)	0.010
Sex			<0.001			
M	2678 (50.30)	346 (40.05)		48.88	49.33	0.013
F	2646 (49.70)	518 (59.95)		51.12	50.67	-0.012
BMI	23.32 (20.66 to 26.40)	23.44 (20.93 to 26.57)	0.302	23.31 (20.63 to 26.38)	23.53 (20.94 to 26.61)	-0.009
Triage			0.035			
1	183 (3.44)	18 (2.08)		3.25	3.27	0.010
2	1361 (25.56)	193 (22.34)		25.11	25.12	0.000
3	3740 (70.25)	644 (74.54)		70.85	70.84	-0.000
4	39 (0.73)	9 (1.04)		0.78	0.77	-0.007
5	1 (0.02)	0 (0.00)		0.01	0.00	-0.255
HR	94.00 (81.00 to 109.0)	92.00 (78.50 to 107.0)	0.002	94.00 (81.00 to 109.0)	92.00 (78.00 to 107.0)	-0.050
SBP	127.0 (112.0 to 147.0)	128.0 (112.0 to 147.0)	0.695	127.0 (112.0 to 147.0)	129.0 (112.0 to 148.0)	0.023
DBP	78.00 (70.00 to 86.00)	78.00 (70.00 to 86.00)	0.717	78.00 (70.00 to 86.00)	78.00 (70.00 to 87.00)	0.001
BT	36.80 (36.30 to 37.40)	36.70 (36.20 to 37.20)	0.008	36.80 (36.30 to 37.40)	36.70 (36.20 to 37.20)	-0.056
RR	20.00 (19.00 to 20.00)	20.00 (19.00 to 20.00)	0.857	20.00 (19.00 to 20.00)	20.00 (19.00 to 20.00)	0.012

Comorbidities	3896 (73.18)	588 (68.06)	0.001	72.48	73.08	0.011
ED resource utilization						
LOS in ED (hr)	11.10 (6.40 to 20.80)	12.80 (7.85 to 21.20)	<0.001	11.10 (6.40 to 20.80)	13.10 (8.00 to 21.70)	0.058
ED cost (NTD)	11610.5 (8346.5 to 14645.5)	11796.0 (8225.0 to 14966.5)	0.879	11590.0 (8328.0 to 14599.0)	12034.0 (8414.0 to 15369.0)	0.038
CT	3778 (70.96)	588 (68.06)	0.082	71.00	68.23	0.056

Data are presented as frequency (percentage). Continuous variables are presented as Median (Interquartile Range, IQR) to account for the non-normal distribution of weighted data. E value =1.46

IPTW: inverse probability of treatment weighting; SMD: Standardized mean difference, with values <0.1 indicating negligible imbalance and 0.1–0.2 suggesting minimal imbalance; PoCUS: point of care ultrasonography; CT: CT only; LOS: length of stay; ED: emergency department; NTD: New Taiwan Dollars

Supplementary Table S2. Sensitivity analysis of ED length of stay comparing the excluded 'PoCUS > 1 hour' cohort (N=864) and the 'No PoCUS' cohort (N=5,324) before and after IPTW adjustment.

	**Data before IPTW**			**Data after IPTW**		
Outcome measures, RM (95% CI)	No- PoCUS	PoCUS >1 hour	p-value	PoCUS > 1 hour	absolute difference	p-value
ED LOS	Ref.	1.11 (1.05 to 1.18)	<0.001	1.11 (1.07 to 1.15)	1.77(hr)	<0.001

Adjusted for age, gender, triage, BMI, and comorbidities.

Data are presented as Gamma distribution with log link (handles right-skewed data). RM: ratio of means; OR: odds ratio; IPTW: inverse probability of treatment weighting; PoCUS: point of care ultrasonography.
